# Recombinant phospholipase A_2_ inhibitor of *Sinonatrix annularis* (ringed water snake) attenuates hemorrhagic action of the venom from *Daboia siamensis* (Siamese Russell’s viper)

**DOI:** 10.2478/abm-2025-0038

**Published:** 2025-12-31

**Authors:** Wichit Thaveekarn, Jureeporn Noiphrom, Asada Leelahavanichkul, Orawan Khow

**Affiliations:** Queen Saovabha Memorial Institute, Thai Red Cross Society, Bangkok 10330, Thailand; Department of Medicine, Faculty of Medicine, Chulalongkorn University, Bangkok 10330, Thailand; Center of Excellence on Translational Research in Inflammation and Immunology (CETRII), Department of Microbiology, Faculty of Medicine, Chulalongkorn University, Bangkok 10330, Thailand

**Keywords:** *Daboia siamensis*, hemorrhage, phospholipase A_2_, phospholipase A_2_ inhibitor

## Abstract

**Background:**

Phospholipase A_2_ (PLA_2_) is a common enzyme in snake venoms from many species that hydrolyzes phospholipids in cell membranes, causing local and systemic adverse effects. Interestingly, PLA_2_ inhibitors in the snake blood are a natural neutralization against PLA_2_ that is leaked into the circulation. Hence, synthesizing liver PLA_2_ inhibitors might be a novel and effective anti-venom strategy.

**Objective:**

To test if PLA_2_ inhibitor gamma form (PLIϒ) reduces the hemorrhagic effect of crude *Daboia siamensis (D. siamensis*) (a venomous viper with high PLA_2_ abundance in the venom).

**Methods:**

PLIϒ was synthesized and purified from the data of *Sinonatrix annularis*. Then, PLA_2_ activity in the snake venoms was screened and tested against PLA_2_ activity from crude *D. siamensis* venom. The hemorrhage effect of crude D. *siamensis* was measured with the minimum hemorrhagic activity dose in mice with and without PLIϒ.

**Results:**

*D. siamensis* had the highest PLA_2_ activity among the 5 selected venomous snakes. The PLIϒ reduced the PLA_2_ activity of crude *D. siamensis* by up to 34.8% (*in vitro*) and decreased hemorrhagic spots in mice by up to 30.2% on the inner surface of mouse skins compared with controls.

**Conclusion:**

PLIϒ reduced PLA_2_ activity and was effective in mitigating the hemorrhagic effect of crude *D. siamensis*.

Venomous snake bite is still a public health problem in Thailand and other tropical countries, causing acute medical emergencies, including paralysis, bleeding disorders, fatal hemorrhage, kidney failure, and local tissue destruction [1]. The pharmacological effects of snake venoms are classified into hemotoxic, neurotoxic, and cytotoxic types [2] with several dominant enzymes, including phospholipase A_2_ (PLA_2_), metalloproteinases, L-amino acid oxidase, serine proteases, and 3-finger peptides [3]. Among all, PLA_2_, a lipolytic enzyme, can be found in venoms of the snakes in the Elapidae family (the snakes with permanently erect fangs at the front of the mouth) and the Viperidae family (the snakes with long, hinged fangs) with either neurotoxic or myotoxic effects [4]. As such, PLA_2_ was discovered in 1951 based on the hydrolysis of phosphatidylcholine by cobra venom, similar to pancreatic enzymes [5] that are categorized into 5 forms, including secretory, cytosolic, Ca^2+^-independent, platelet-activating factor acetyl hydrolases, and lysosomal PLA_2_ [6]. On snake bite wounds, PLA_2_ intensifies hemorrhagic, cytotoxic, and myotoxic effects [7] through hydrolysis of the ester bond at the Sn-2 position of phospholipids in the plasma membrane of various cells, especially erythrocytes, resulting in hemolysis [8]. Indeed, PLA_2_-induced hemorrhagic effects are mentioned in several venoms, for example, *Trimeresurus mucrosquamatus* [9], *Hypnale hypnale, Echis carinatus*, and *Daboia russelii* [10]. On the other hand, phospholipase A2 inhibitor (PLI) is a self-protective enzyme against the presence of venom toxins in the snake circulatory blood of several families (Elapidae, Viperidae, Hydrophidae, Colubridae, and Boidae) [11]. Unsurprisingly, PLI is considered an ideal candidate for anti-inflammation and anti-venoms [12] with 3 structural types, including alpha (α), beta (β), and gamma (ϒ), and phospholipase A2 inhibitor gamma form (PLIϒ) demonstrates the broadest spectrum of PLA_2_ inhibition [13]. Interestingly, PLIϒ isolated from *Sinonatrix annularis* (ringed water snake), a non-venomous snake, reduces hemorrhagic toxicity of *Deinagkistrodon acutus, Naja atra*, and *Agkistrodon halys* venoms after an injection into the gastrocnemius muscle [14] and subcutaneous sites in mice [15].

Indeed, *Daboia siamensis* (*D. siamensis*) is a common venomous snake found in many parts of Southeast Asia, including Thailand, Vietnam, Cambodia, Laos, and Myanmar. Its venom interferes with blood clots, leading to hemorrhage and internal bleeding. Because *D. siamensis* is a common venomous snake in the Northern and Central parts of Thailand, causing major morbidities (disseminated coagulation, hemolysis, and limb edema) mainly due to PLA_2_ effects [16]. PLIϒ might be an interesting candidate for anti-venom. Indeed, up to 70% of the dry weight of *D. siamensis* venom in some specimens is PLA_2_, which can induce a hemorrhagic effect through vascular endothelial damage [16]. Accordingly, bites from *D. siamensis* (Siamese Russell’s viper) are mostly reported in Thailand with severe local tissue swelling, coagulopathy, nephrotoxicity, and, in rare cases, cardiac manifestations and cerebral infarction [16]. Due to the presence of PLA_2_ in several types of snake venom, the test of PLIϒ against PLA_2_ in the venom of several snakes is interesting. Thus, in this study, we synthesized the recombinant PLIϒ based on the liver tissue data of *S. annularis* (a snake with PLA_2_ resistance) and tested the PLA_2_ enzymatic activities against venoms of several types of snakes in vitro, and evaluated the impacts of PLIϒ in mice injected with crude *D. siamensis* venom. We hypothesized that PLA_2_ inhibitors might be an adjunctive or an alternative treatment of snakebites in the near future.

## Materials and methods

### Preparation and purification of PLIϒ

The *S. annularis* PLIϒ gene, a partial sequence without signal peptides (553 bp, JN975878, GenBank) from the snake liver, was processed into the pET-24a vector by Synbio Technologies. As such, *Escherichia coli* (*E. coli*) BL21 (DE3) cells, the bacterium with compatibility with pET24a expression allow isopropyl β-D-1-thiogalactopyranoside (IPTG) to release the Lac repressor and initiate RNA polymerase binding to the promoter for gene transcription, were transformed with pET24a-PLIϒ of *S. annularis* by heat shock on Luria Bertani (LB) medium containing 50 μg kanamycin agar plate at 37°C. Notably, the pET-24a (+) vector is a popular plasmid vector used for recombinant protein expression in *E. coli* with a strong T7 promoter allowing high expression of target proteins, Lac operon control for inducing the expression with IPTG, and a simple 6x polyhistidine-tag (His-Tag) for protein purification. Each colony that grew on a kanamycin agar plate was picked by needle tip, transferred to a polymerase chain reaction (PCR) reagent tube for *PLIϒ* gene amplification, and confirmed as a positive colony by PCR. Forward primer: TAATACGACTCACTATAGGG, reverse primer: GCTAGTTATTGCTCAGCGG, and *TaKaRa Taq* DNA polymerase (Takara Bio Inc.) were used in the PCR. The PCR conditions were 5 min at 94 °C, 35 cycles of 3 min at 94 °C, 30 sec at 55 °C, and 30 sec at 72 °C, followed by an extension period of 7 min at 72 °C (Perkin Elmer). The obtained PCR product size of around 500 bp would confirm a positive clone colony. The positive colony was incubated in 50 mL of LB broth containing 50 μg kanamycin and shaken at 37 °C, overnight. Then, 10 mL of cell culture was transferred into 1 L of LB containing 50 μg kanamycin. The cells were grown at 37 °C with shaking at 160 rpm. When the optical density (OD) at 600 nm reached 0.4-0.6, IPTG was added to the culture to a final concentration of 1 mM and shaken at 37 °C, overnight. After that, PLIϒ expression was detected by 1 mL of the cell suspension to run 10% sodium dodecyl sulfate-polyacrylamide gel electrophoresis (SDS-PAGE). One liter of cultivated recombinant BL21 (DE3) cells was washed with phosphate-buffered saline (PBS) buffer, pH 7.4. The recombinant bacteria were resuspended in 20 mL PBS buffer, pH 7.4, and rested on ice for 20 min. The cells were sonicated on an ice bath with a program of 10 s sonication and stopped by 2 min intervals of resting for 4 rounds. After centrifugation of the lysate at 6,000 rpm for 10 min, the pelleted cell (part of the inclusion bodies) was collected. The pelleted cell was dissolved in 8 M urea, 20 mM Na_2_HPO_4_, and 0.5 M NaCl, pH 7.4, and gently agitated with a magnetic bar at 4 °C overnight. The undissolved particles were removed by centrifugation, and the protein concentration of the supernatant was determined by Qubit Protein Assay Kits, Thermo Fisher. The PLIϒ was purified by an *ÄKTA pure* machine (GE) based on the His-tag method that typically consists of at least 6 histidine (His) residues at the N- or C-terminus of the protein. The supernatant was loaded onto a 1 mL HisTrap FF column (Cytiva), which was pre-equilibrated with 5-fold column volumes (CV) of binding buffer (8 M urea, 20 mM Na_2_HPO_4_, and 0.5 M NaCl, pH 7.4). The column was continually washed with binding buffer to baseline, then washed with a urea gradient for refolding from 8 M to 0 M urea by creating a stepwise gradient from 100% of urea to 0% of urea solution (20 CV, 20 mL) with a solution containing 8 M urea, 20 mM NaH_2_PO_4_, 20 mM, 0.5 M NaCl, pH 7.4, and 20 mM NaH_2_PO_4_, 20 mM, 0.5 M NaCl, pH 7.4. Finally, the column was washed by a stepwise gradient of elution buffer 10% elution buffer containing 500 imidazole, 20 mM NaH_2_PO_4_, 20 mM, 0.5 M NaCl, pH 7.4 to elute histidine-tag PLIϒ (His6-PLIϒ). After that, PLIϒ was dialyzed with 0.98% normal saline, and protein concentration was measured using Qubit Protein Assay Kits (Thermo Fisher Scientific).

### Identification of protein by mass spectrometry

The protein solution was digested using 12.5 μg/mL of sequencing-grade trypsin (Promega) and incubated at 37 °C for approximately 16 h. The supernatants were then transferred to a new tube, and 100 μL of 50% acetonitrile (ACN)/0.5% formic acid was added. The mixtures were finally dried with a speed vac, and the resulting peptides were suspended in 10 μL of 50% ACN/0.1% formic acid. The peptides were then analyzed by liquid chromatography-mass spectrometer (LC-MS/MS; TripleTOF 6600+, Sciex) at Mahidol University (Frontier Research Facility), Bangkok, Thailand. Protein identification was performed using the search engine named PEAKS (Version: PEAKS Studio 10.6 build 20201221).

### PLA_2_ activity and the attenuation by PLIϒ(in vitro)

The assay was performed following a previous publication [17]. Briefly, the crude venoms (powdered) of *D. siamensis, Trimeresurus albolabris (T. albolabris), Calloselasma rhodostoma (C. rhodostoma), Naja kaouthia (N. kaouthia*), and *Ophiophagus hannah (O. hannah*) were obtained from the snake farm (Queen Saovabha Memorial Institute, Bangkok), and 25 μL of 0.5 mg/mL of crude snake venom in PBS, pH 7.4, was added to 500 μL of 10 mM Tris-HCl, 10 mM CaCl2, and 0.1 M NaCl, pH 8.0, on ice. Then, 50 μL of 4-nitro-3-octanoyloxy benzoic acid (Abcam), a chromogenic substrate for PLA_2_, was added and incubated at 37 °C for 20 min before stopping the reaction by 50 μL of stopper buffer (2.5% Triton X-100) on ice for 5 s. The reaction tubes were left at room temperature for 5–10 min and measured for OD at 425 nm by a spectrophotometer (Beckman, Brea). The specific activity of PLA_2_ is defined as a change in absorbance of 0.1 AU at 425 nm, equivalent to 25.8 nanomoles of released chromophore. To explore the neutralization property of PLIϒ, 25 μL of purified PLIϒ in concentrations of 10 μg, 20 μg, 30 μg, 40 μg, and 50 μg were independently added into the crude *D. siamensis* (25 μL of 0.5 mg/mL) and incubated at room temperature for 30 min before testing PLA_2_ activity using the protocol mentioned earlier.

### Animal and animal model

The study protocol was approved by the Institutional Animal Care and Use Committee of the Queen Saovabha Memorial Institute, Bangkok, Thailand (Certificate of approval no. QSMI-ACUC-06-2025), following the National Institutes of Health (NIH), USA. Male MLAC/ICR mice (Institute of Cancer Research strain), obtained from the Microbiological and Laboratory Animal Center (MLAC), 8 weeks of age, weighing 20 ±5 g from Nomura Siam International Co., Ltd. (Lumphini, Pathumwan, Bangkok, Thailand) were used. Notably, only male mice were used to reduce the gender variability in the proof of concept experiments, and the impacts on female mice need further testing. All experiments were performed in a standard facility of rodent housing with a temperature-controlled environment (24 ± 2 °C), 50% relative humidity, a 12 h light-dark cycle (light from 7:00 a.m. to 7:00 p.m.), and a standard diet and water. To test the MHD of *D. siamensis venom, the* hemorrhagic assay, following a previous description [18], was performed. Briefly, crude *D. siamensis* venom in 50 μL PBS was intradermally injected (ID) in mice with 0 μg, 4 μg, 8 μg, 12 μg, and 16 μg (n = 5/dose). The mice with PBS alone (0 μg of venom) were used as a negative control. After 3 h, mice were euthanized using CO_2_, and the dorsal patch of skin was removed to observe for hemorrhagic lesions on the inner surface of the skin (measured by cross-diameter hemorrhagic spots).

The diameter of the hemorrhagic spot on the inner surface of the mouse skin was measured to determine the MHD, defined as the venom dose that produces a 10-mm hemorrhagic lesion. To test against the hemorrhagic *activity of D. siamensis*, PLIϒ (10 μg in 25 μL) was mixed with 25 μL of 16 μg of *crude D. siamensis venom* and incubated at room temperature for 30 min before using this mixed solution (50 μL) to ID into the mice (n = 5). The group of mice that received PLIϒ alone was used as a negative control (n = 3). After 3 h, the mice were euthanized using CO_2_ and the dorsal patch of the skin was removed and observed for hemorrhage on the inner surface of the skin as mentioned above. The spot *(mm*) of the hemorrhagic area on the inner surface of the skin was measured as the representative hemorrhagic activity.

### Statistical analysis

Data presented by mean standard error (SE) and tested by 1-way analysis of variance followed by Bonferroni’s multiple comparisons tests using PRIMER of Biostatistics software analysis. The differences were considered significant at *P* < 0.05.

## Results

### Gene expression and purification of PLIϒ

**Figure 1.**
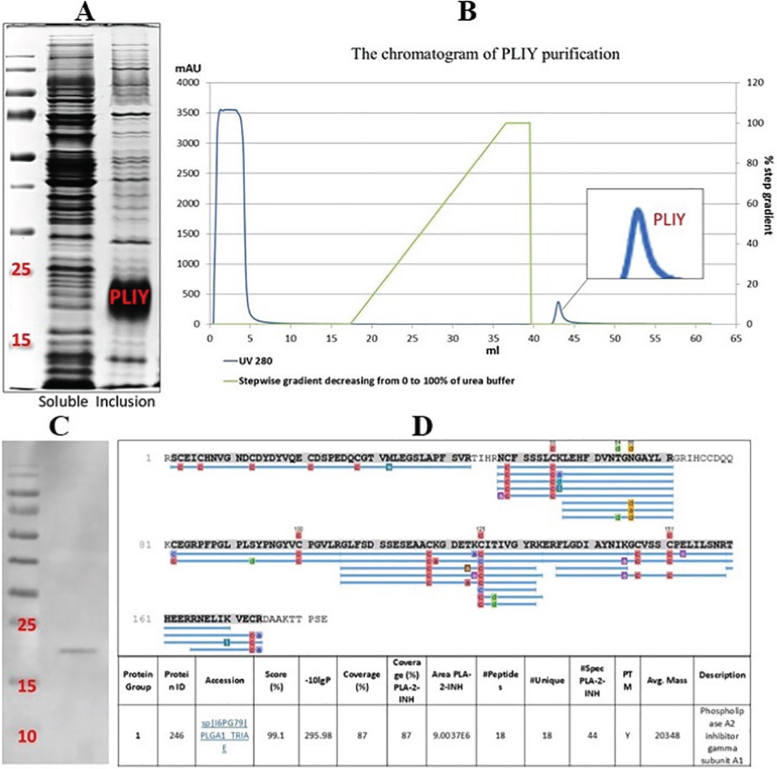
The representative picture of SDS-PAGE indicated the expression of PLIϒ proteins in the soluble (left side) and inclusion forms (right side) **(A)** was demonstrated. The chromatogram of PLIϒ after the purification by the HisTrap FF column **(B)** and the PLIϒ protein isolated from the column (the magnified peak with the red-colored alphabets in **B**) was tested for the purification as indicated in SDS-PAGE **(C)**. The amino acid sequence of isolated PLIϒ, as performed by LC–MS/MS, was also shown **(D)**. LC–MS/MS, liquid chromatography-mass spectrometry; PLIϒ, phospholipase A_2_ inhibitor gamma form; SDS-PAGE, sodium dodecyl sulfate-polyacrylamide gel electrophoresis.

The *PLIϒ* gene was inserted into the pET-24a vector and cultured (see method). The expression of PLIϒ protein (Figure 1A), the chromatogram of purification (Figure 1B), and purified PLIϒ were demonstrated on the SDS-PAGE (Figure 1C) and confirmed by mass spectrometry (Figure 1D).

The *PLIϒ* gene was inserted into the pET-24a vector, and the cell culture was activated by IPTG, the inducer of protein expression. Then, the cells were harvested, disrupted by sonication, and processed on the SDS-PAGE to detect PLIϒ in the soluble or inclusion forms. There were highly expressed PLIϒ proteins in the inclusion bodies with the smeared band between 15 kDa and 25 kDa (Figure 1A).

Notably, protein expressions in inclusion bodies could be found in recombinant proteins through the accumulation of misfolded proteins in the cytoplasm or periplasm of the host cells, and the refolding of the proteins is naturally possible [19]. Then, a chaotropic agent (8 M urea) was used to recover and refold PLIϒ protein from inclusion bodies to its natural form during purification (see method). As such, PLIϒ in 8 M urea was loaded into a HisTrap FF column (1 mL) and was refolded by a stepwise gradient. In the refolding step (16–40 mL), the urea concentration gradually reduced from 8 M to 0 M (no urea buffer in the column) without noise protein (no detected UV 280 signal), which process was under the green line area. The percentage of gradient urea from 100 to 0 in the green line was related to graph axis 3 on the right (Figure 1B). After the folding step, the PLIϒ peak gradually appeared at mL of 42–45 with a stepwise gradient of 10% of imidazole elute buffer. After finishing PLIϒ purification, a chromatogram was performed on only the single peak (blue-colored peak), representing the purified PLIϒ (redcolored alphabet) without other contaminated protein peaks (Figure 1B). The high protein peak at the front part of the chromatogram (at mL 0–10) was the unbinding proteins to the HisTrap FF column. Then, the purification was performed using a ready-to-use column, prepacked with the pre-charged Ni Sepharose 6 Fast Flow for preparative purification of His-tag recombinant proteins by immobilized metal ion affinity chromatography (see method). As such, PLIϒ obtained from the single peak showed a single protein band at approximately 20 kDa on SDS-PAGE (Figure 1C). Before purification, the dissolved pellet cell (from 1 L of culture) in 8 M urea had a concentration of 4.44 mg/mL. Then, after the purification by an *ÄKTA pure* machine (GE), the PLIϒ protein concentration was 187.20 μg/mL (4% of total protein).

### PLA_2_ activity and the attenuation of D. siamensis venom by PLIϒ

**Figure 2.**
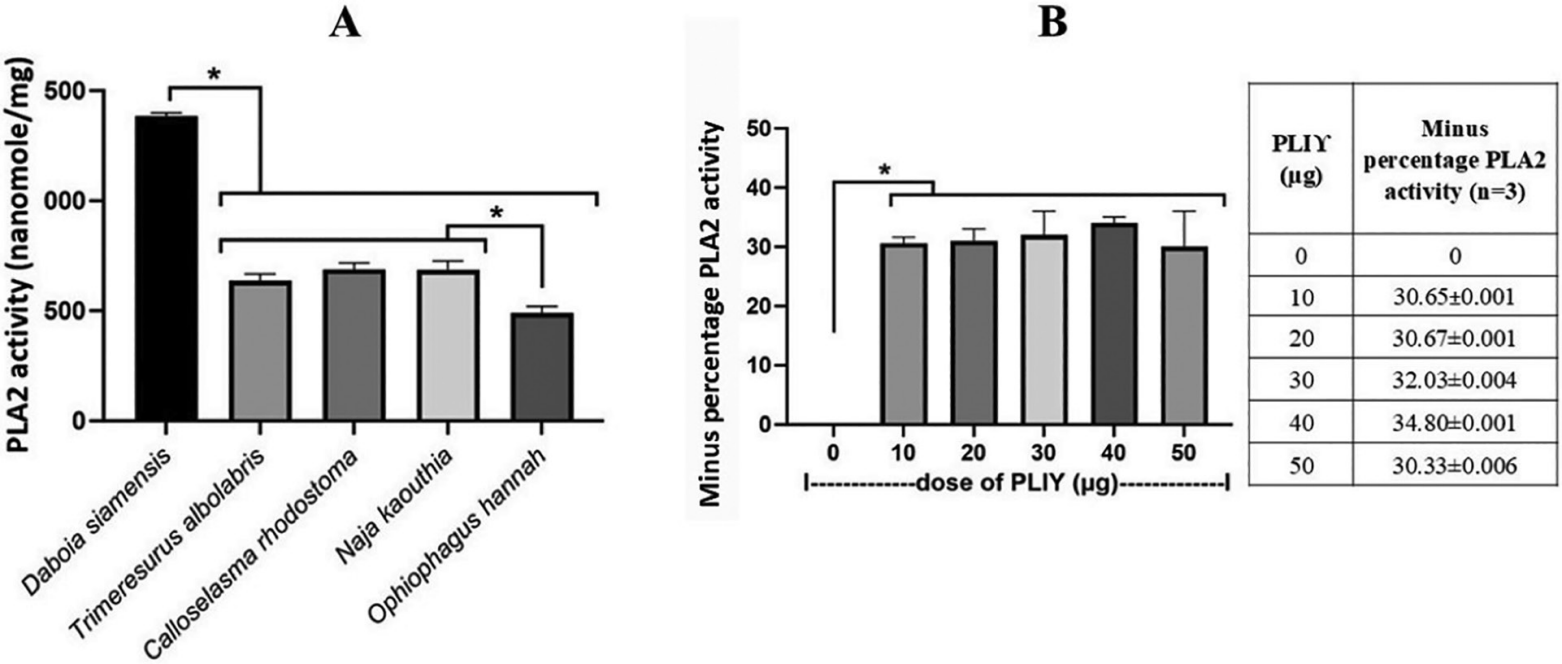
The screening PLA_2_ activity of venoms from several types of snakes **(A)** and the minus percent of the PLA_2_ activity in *D. siamensis* venom (a representative venom) by PLIϒ **(B)** was demonstrated. Isolated triplicate experiments were performed. PLA_2_, phospholipase A_2_; PLIϒ, phospholipase A_2_ inhibitor gamma form.

**Figure 3.**
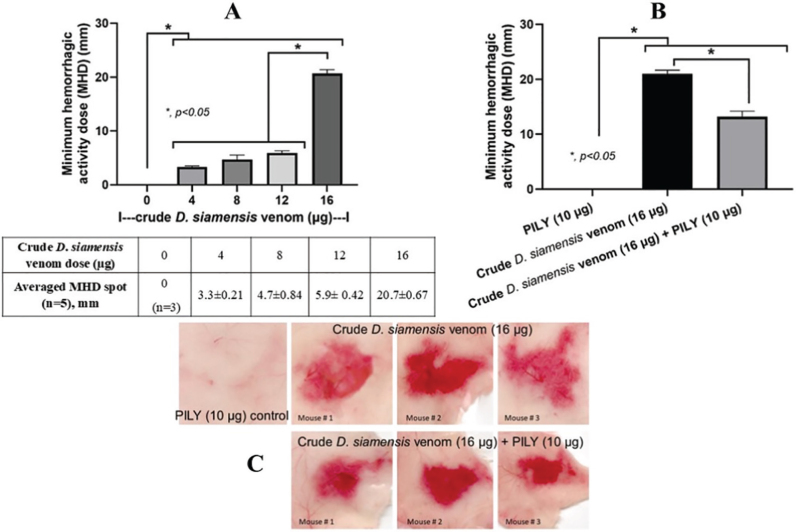
Crude *D. siamensis*-induced hemorrhagic activities with MHD without or with PLIϒ **(A, B)** with the representative pictures of mouse skin **(C)** were demonstrated (n = 3–5/group). MHD, minimal hemorrhagic dose; PLIϒ, phospholipase A_2_ inhibitor gamma form.

Five snake venoms, including *D. siamensis, C. rhodostoma, N. kaouthia, T. albolabris*, and *O. hannah*, were screened for PLA_2_ activity (Figure 2A) and tested by the recombinant PLIϒ from the previous experiments (Figure 2B). As such, the highest PLA_2_ activity was presented in *D. siamensis* (Siamese Russell’s viper), and the lowest level was in *O. hannah* (king cobra) (Figure 2A). Due to the highest PLA_2_ activity in crude *D. siamensis* venom, the PLA_2_ inhibitory activity of PLIϒ was tested in *D. siamensis* venom as a representative experiment. As such, PLIϒ at several concentrations (10–50 μg) similarly attenuated PLA_2_ activity (Figure 2B). To test the activity in vivo, mice were injected intradermally with different doses (0–16 μg) of crude venom from *D. siamensis* to determine the emergence of MHD in the diameter (mm) of the inner skin of mice. As such, the intradermal injection of crude venom from *D. siamensis* at the dose of 16 μg demonstrated the most severe lesion at 3 h post-injection compared to other doses, as indicated by the diameter of hemorrhagic spots on the inner side of the mouse’s skin (Figure 3A). Despite the severe hemorrhage caused by 16 μg *D. siamensis* venom, mixing PLIϒ (10 μg) into the venom before injection induced less severe hemorrhage, as indicated by lower MHD values (Figures 3B, C), suggesting the neutralizing effect of PLIϒ. Notably, only PLIϒ at 10 μg was used in Figure 3 due to the non-dosedependent effect among 10–50 μg in Figure 2B. Our data demonstrated a proof-of-concept to use PLIϒ against *D. siamensis* venom; the impacts of PLIϒ against other snake venoms will be interesting.

## Discussion

PLA_2_, a common enzyme in several snake venoms, can damage cell membranes, cause tissue inflammation, and induce severe inflammation in snakebite wounds. It is a major component enzyme of *D. siamensis* venom. However, a PLIϒ, especially from *S. annularis*, mitigated the action of hemorrhage and myonecrosis caused by PLA_2_ in snake venom [14]. In this study, the PLIϒ gene was cloned into the pET-24a vector for recombinant expression. The His-tagged PLIϒ protein was purified using a HisTrap FF nickel-affinity column, which selectively binds polyhistidine-tagged proteins while minimizing non-specific adsorption. The purified protein eluted as a single peak and appeared as a single band of approximately 20 kDa on SDS-PAGE (10%), with its identity confirmed by LC–MS/MS. After purification, the final yield of renatured PLIϒ protein was low, 4% (187.2 μg/mL) of the total protein (4.4 mg/mL), before entering the HisTrap FF column. Additionally, recombinant PLI from *S. annularis* and natural PLI gamma protein from the serum of *S. annularis* demonstrate good inhibitory activity against *D. acutus, N. atra*, and *A. halys* hemorrhagic toxicity; however, natural PLI gamma shows a better result [15]. Hence, post-translational modifications were important for the expression system, and the *E. coli* expression system demonstrated limited post-translational modifications, such as glycosylation. Further studies to improve protein synthesis are needed.

The abundance of PLA_2_ relative to the total venom weight of *Daboia* snakes (subfamily Viperidae with several proteases and PLA_2_ in venom) in Pakistan (63.8%) [20], Indonesia 48.4%, and Thailand 37.9% [21]. In Viperidae snake venom, PLA_2_ could be found at approximately 32%–59.8% of the total weight of the venom [22]. The amount of PLA_2_ was differentially expressed depending on locality [23] and may be attributed to adaptive evolution. Here, *D. siamensis* showed the highest PLA_2_ among 5 crude snake venoms with PLA_2_ activity in Thailand. The PLA_2_ activity of *O. hannah* was the lowest. Possibly due to the lowest PLA_2_ abundance (3%–4%) from proteome analysis, resulting in less severe inflammatory, lethal, and cardiotoxicity in mice [24]. To demonstrate PLA_2_ activity in *D. siamensis* venom by PLIϒ, 10–50 μg of purified PLIϒ similarly neutralized crude *D. siamensis*, implying a non-dose-dependent effect, and the lowest dose (10 μg) might have the least adverse effect with an acceptable therapeutic action. For the MHD, the dose of venom or toxin that induces a hemorrhagic spot of 10 mm diameter, 16 μg of crude *D. siamensis* induced 20.7 mm. While the lower doses (0 μg, 4 μg, 8 μg, and 12 μg) showed too small spots. Interestingly, recombinant PLIϒ from *S. annularis* could reduce PLA_2_ activity in *D. siamensis* venom in mice (reduced MHD), even though the reduced PLA_2_ activity *in vitro* was only up to 34.8%. Lower roll pictures represented 3 out of 5 images of crude *D. siamensis* venom-induced hemorrhage spots that PLIϒ partially neutralized. These demonstrated that PLIϒ could reduce hemorrhagic spots caused by *D. siamensis*.

However, snake PLA_2_ inhibitors act on PLA_2_s through unclear mechanisms. Some studies suggested that the PLI_α_/PLA_2_ binding site was related to the carbohydrate recognition domain region of the molecule, which recognizes and binds to the enzyme, preventing its toxic activity. Indeed, PLI_α_ and AnMI isolated from the plasma of *Atropoides nummifer* snakes demonstrate 67.0% reduced toxic activities of myotoxin II and 93.0% reduced action of myotoxin B [25]. Accordingly, PLIβs inhibit only the basic group II PLA_2_ (inflammatory inducer), while PLIγs inhibit PLA_2_ from groups I, II, and III. The mechanism of PLI depends on the type and the specific enzyme targets of PLA_2_. Hence, PLIs have potentially reduced tissue damage and systemic effects from the envenomation. However, there are some limitations in clinical settings, such as the challenging process of obtaining PLA_2_ inhibitors from snake venom (complex purification process and limited availability), and the difficult large-scale production. Although several recombinant techniques are used to produce PLA_2_ inhibitors, the correct protein folding and their functions of these proteins remain a challenge. Despite promising results in animal studies, clinical trials are needed to evaluate their potential use. PLIs might be applied to mitigate the effects of venom, enhance the treatment of snakebites, and improve clinical outcomes; however, more research is necessary.

In conclusion, PLIϒ recombinant from *S. annularis* could reduce PLA_2_ activity and mitigate hemorrhage of crude *D. siamensis in vivo*. Our results supported the ability of PLIϒ as a candidate for anti-venom. Further studies to explore PLIϒ against other snake venoms are interesting.
